# Patterns of Urine Testing and Antibiotic Use for Atypical UTI Symptoms in Cognitively Impaired Long-Term Care Residents: A Retrospective Study of Diagnostic Criteria Adherence

**DOI:** 10.7759/cureus.90422

**Published:** 2025-08-18

**Authors:** Kaku Kuroda, Alissa N Nizinski, Kaitlin Kyi, Samuel K Ayo, Erin Kirkpatrick, Ian M Deutchki, Dallas L Nelson

**Affiliations:** 1 General Medicine, University of Toyama, Toyama, JPN; 2 Geriatrics, University of Rochester, Rochester, USA; 3 Oncology, Rogel Cancer Center, Ann Arbor, USA; 4 Geriatrics and Family Medicine, University of Rochester, Rochester, USA

**Keywords:** asymptomatic bacteriuria, cognitive impairment, geriatric medicine, long-term care, urinary tract infection

## Abstract

Background: Diagnosing urinary tract infection (UTI) in patients with impaired communication is challenging due to reliance on subjective symptoms. This study compared diagnostic criteria fulfillment, the results of urine testing, and antibiotic use for atypical UTI symptoms between verbal patients and those with impaired communication in a long-term care (LTC) setting.

Methods: In this cross-sectional study, we retrospectively analyzed 127 urinalyses and urine cultures ordered between January 1 and December 31, 2023, from 83 patients in an LTC facility in New York, USA. Data included demographics, comorbidities, reasons for urinalysis, results, and antibiotic use. We assessed the adherence to Loeb's diagnostic and treatment criteria and the updated McGeer criteria.

Results: Patient background characteristics did not differ significantly between groups. Among non-catheterized patients, verbal patients were significantly more likely to meet the Loeb diagnostic criteria (54.7% vs 20.8%, p=0.005), Loeb treatment criteria (42.2% vs 12.5%, p=0.009), and updated McGeer criteria (50.0% vs 20.8%, p=0.014), while rates of pyuria (90.5% vs 83.3%, p=0.435) and positive urine culture (75.0% vs 66.7%, p=0.434) did not differ. These findings suggest a higher prevalence of asymptomatic bacteriuria in patients with impaired communication. Among catheterized patients, there were no significant differences between groups in meeting diagnostic criteria. Antibiotics were prescribed for over 90% of patients in both groups whose urine samples were collected.

Conclusions: Patients with impaired communication are less likely to meet established UTI diagnostic criteria despite similar urine test positivity, indicating possible overdiagnosis and overtreatment. Further research is needed to develop and implement a modified diagnostic algorithm tailored to the unique clinical presentation of LTC residents with impaired communication. High antibiotic prescription rates emphasize the need to reduce unnecessary tests to minimize inappropriate antibiotic use.

## Introduction

Differentiating between a urinary tract infection (UTI) and asymptomatic bacteriuria and deciding when to initiate antibiotic treatment within long-term care (LTC) settings presents challenges due to the absence of definitive clinical and laboratory standards for commencing antibiotics, alongside the high prevalence of cognitive impairment in LTC units [1]. Based on existing evidence, ordering urinalysis and antimicrobial therapy is not recommended for individuals exhibiting isolated nonspecific signs or noninfectious symptoms such as fatigue or delirium [2]. Atypical symptoms of UTI may include altered mental status, changes in urine odor or color, nonspecific abdominal discomfort, leukocytosis, reduced oral intake, and gastrointestinal symptoms such as nausea or diarrhea. ​​​A cross-sectional study investigating 651 older individuals in nursing homes suggested that urine culture was of little or no value in clarifying the etiology of diffuse symptoms, including restlessness, fatigue, confusion, aggressiveness, or not being herself/himself [3]. Several consensus-based criteria for diagnosing UTI have been published, but these criteria often vary [4]. This diagnostic uncertainty may contribute to the overuse of antimicrobials and, subsequently, the emergence of antimicrobial resistance.

The Loeb diagnostic criteria are commonly used to determine whether to order a urine culture [5]. Patients who do not have an indwelling catheter should have acute dysuria or fever (>37.9°C or a 1.5°C increase above baseline temperature) and at least one of the following: new or worsening urgency, frequency, suprapubic pain, gross hematuria, costovertebral angle tenderness, or urinary incontinence [5]. However, assessing this criterion is often difficult for patients with cognitive impairment who cannot accurately complain of UTI symptoms unless they have objective findings such as gross hematuria or costovertebral angle tenderness. 

Several proposed algorithms have been published to assess when to order urinalysis [6-8]. Nevertheless, these algorithms include subjective symptoms such as dysuria; thus, it is difficult to determine the suitability of ordering urinalysis and urine culture for patients with cognitive impairment. Within these criteria, the recommendation for conducting urinalysis or urine culture in cognitively impaired patients who cannot explain UTI-related symptoms accurately is unclear. Our objective was to compare data on clinical decision-making and the outcomes of urinalysis and urine culture ordered for verbal patients and those with impaired communication and assess patterns of urine testing and antibiotic use for atypical UTI symptoms in cognitively impaired LTC residents, with a focus on adherence to established diagnostic and treatment criteria.

## Materials and methods

Study design

This was a retrospective, cross-sectional study conducted at the Monroe Community Hospital, an LTC facility located in Rochester, New York, USA. The facility has approximately 350 beds and features specialized units that provide care for individuals with dementia and respiratory services. Patients of all ages are included in the study, particularly those admitted to the sub-acute rehabilitation unit and the respiratory care unit.

Inclusion and exclusion criteria

We included all residents of the Monroe Community Hospital who had at least one urinalysis or urine culture ordered between January 1, 2023, and December 31, 2023, with the availability of complete clinical and laboratory data for the episode. We excluded duplicate tests from the same episode, retaining only the first set of tests per episode. In addition, urine tests ordered for patients who met the 100-100-100 early detection tool criteria [9], despite the absence of UTI symptoms, were excluded to avoid confounding effects related to sepsis screening protocols. This tool was chosen over the systemic inflammatory response syndrome (SIRS) and the quick sepsis-related organ failure assessment (qSOFA) criteria due to its higher sensitivity in nursing home settings [9]. Furthermore, qSOFA was not recommended as a single screening tool in the most recent sepsis guideline [10]. Conversely, if residents did not meet the 100-100-100 criteria, all urine tests ordered for symptoms and signs not included in the Loeb criteria (e.g., isolated fever) were included in the analysis to assess the outcomes of urine testing for atypical presentations of UTI. 

Sampling technique

We used total population sampling (non-probability; no random selection was involved), including all eligible residents meeting the above criteria within the study period. No formal sample size calculation was performed due to the retrospective nature of the study.

Data collection and variables

Data were extracted from the facility’s electronic medical records by two of the authors independently (KK and EK). Collected variables include demographic information (age and sex); clinical status (presence of an indwelling urethral catheter, ambulatory ability, and verbal communication capacity); clinical indications (documented reason for performing urinalysis); laboratory findings (presence of WBCs and bacteriuria on urinalysis and urine culture results); and treatment data (initiation of antibiotic therapy). While inter-rater reliability was not formally calculated, the reviewers used a standardized protocol, and discrepancies were resolved through discussion.

Urinalysis was performed using standard automated dipstick and microscopy techniques available at the facility. Urine culture specimens were processed by the affiliated clinical microbiology laboratory using the standard streak plate method on appropriate culture media. Cultures were incubated and interpreted according to Clinical and Laboratory Standards Institute (CLSI) guidelines, and bacterial growth was quantified in colony-forming units (CFU) per milliliter. Urine tests were ordered by physicians or nurse practitioners, and specimens were collected by nursing staff with training in appropriate sample collection techniques. All of these staff members knew the patients on their assigned floor well.

Patients with impaired communication were defined based on clinical assessments and consensus among care team members. Specifically, patients met this definition if they had any of the following: a Glasgow coma scale (GCS) verbal response score of ≤3, a clinical dementia rating (CDR) scale score of ≥2, or a documented diagnosis of aphasia affecting expressive or receptive language. In addition, for all cases, a clinical consensus was reached through discussion among a physician, a nurse practitioner, and a registered nurse familiar with the patient’s condition to determine whether the patient was able to communicate reliably or not.

We assessed whether urine cultures were obtained per the Loeb minimum criteria for ordering a urine culture (Loeb diagnostic criteria) and whether antibiotic treatment was initiated in line with the Loeb criteria for initiating antibiotics (Loeb treatment criteria). We also applied the updated McGeer criteria to evaluate diagnostic accuracy retrospectively. 

Statistical analysis

Descriptive statistics were used to summarize resident characteristics and test outcomes. Categorical variables were compared using the chi-square (χ²) test, while continuous variables were analyzed using the independent samples t-test. A p-value of <0.05 was considered statistically significant. All analyses were performed using SPSS Statistics version 29 (IBM Corp., Armonk, NY, USA).

Ethical considerations

This study used de-identified data collected retrospectively from existing clinical records. The study protocol was reviewed and approved by the Institutional Review Board (IRB) at the University of Rochester (approval no. STUDY00009436). The requirement for informed consent was waived due to the retrospective nature and anonymization of the data.

## Results

A total of 126 urinalyses and urine cultures were evaluated, encompassing data from 83 patients. Table 1 outlines the fundamental characteristics of these 83 patients. Among them, 22 patients utilized indwelling catheters, while 27 patients had impaired communication due to various cognitive impairments such as aphasia (13 (48.2%) resulting from stroke, brain tumor, or traumatic brain injury), dementia (eight (29.6%)), intellectual disability (four (14.8%)), and psychiatric disorders (two (7.4%)).

**Table 1:** Patient characteristics * Mean±standard deviation

Characteristics		Values (Total n=83)
Age		66.5±15.8*
Sex	Male	41 (49.4%)
	Female	42 (50.6%)
Indwelling catheter	Using	22 (26.5%)
	Not using	61 (73.5%)
Mobility	Ambulatory	24 (28.9%)
	Non-ambulatory	59 (71.1%)
Communication	Verbal	56 (67.5%)
	Impaired	27 (32.5%)

Table 2 presents a cross-tabulation of characteristics between verbal patients and those with impaired communication. No discernible differences in patient background were observed between verbal patients and those with impaired communication. 

**Table 2:** Comparison of characteristics between verbal patients and patients with impaired communication An independent samples t-test was used for age. Chi-square (χ²) tests were used for categorical variables.

Variables	Verbal patients (n=56)	Patients with impaired communication (n=27)	Test statistic	p-value
Age (mean±SD*)	66.4±15.7	66.8±16.1	t(81)=0.114	0.910
Sex: Male	27	14	χ²(1) = 0.096	0.756
Sex: Female	29	13		
Indwelling catheter: Using	16	6	χ²(1) = 0.377	0.539
Indwelling catheter: Not using	40	21		
Mobility: Ambulatory	17	7	χ²(1) = 0.174	0.677
Mobility: Non-ambulatory	39	20		

Table 3 shows symptoms and findings that prompted the order of a urinalysis and urine culture. Of 123 urine tests (both urinalysis and urine cultures), symptoms and findings prompted urine tests that met the Loeb diagnostic criteria in 48 urine tests. These symptoms and findings were seen more frequently in verbal patients than in patients with impaired communication. Other UTI symptoms, including suprapubic localized pain, urinary frequency, hematuria, shaking chills, and Foley obstruction, were also seen more frequently in verbal patients. In contrast, objective findings such as fever or leukocytosis were more often seen in patients with impaired communication. 

**Table 3:** Symptoms or findings that prompted the order of a urinalysis and urine culture * Incomplete data in four cases

Symptoms	Among verbal patients	Among patients with impaired communication
UTI symptoms that met the Loeb diagnostic criteria	42	6
Fever and 1 or more UTI symptoms	2	3
-Dysuria only	15	2
-Dysuria + 1 or more UTI symptoms	19	1
-2 UTI symptoms without dysuria	6	0
Other UTI symptoms (not meeting the Loeb diagnostic criteria)	23	12
-Hematuria	9	2
-Suprapubic pain (localized)	3	2
-Urinary frequency	2	0
-Urinary urgency	2	0
-Shaking chills	1	0
-Fever only	6	7
-Foley obstruction	0	1
Other nonspecific symptoms (atypical UTI symptoms)	23	16
-Altered mental status	6	3
-Change in odor to the color of urine	2	1
-Abdominal pain (nonspecific)	2	2
-Non-UTI symptoms + Leukocytosis	7	3
-Leukocytosis only	2	6
-Poor oral intake	2	0
-Nausea/vomiting	1	0
-Nausea/vomiting/diarrhea	1	0
-Tachypnea	0	1

Table 4 presents the rates of meeting diagnostic criteria, undergoing urine testing, and receiving antibiotics, comparing verbal patients and those with impaired communication without indwelling catheters. Among urine tests of verbal patients, pyuria was present in 57 (90.5%), and urine cultures were positive in 48 (75.0%). However, only 32 (50.0%) met the updated McGeer criteria, suggesting that 25.0% had asymptomatic bacteriuria. In urine tests of patients with impaired communication, urine cultures were positive in 16 (66.7%), while only five (20.8%) met the updated McGeer criteria, indicating that 45.9% may have had asymptomatic bacteriuria. Despite similar background characteristics between the groups, notable gaps were observed in the rates of meeting the three diagnostic criteria. The rates of meeting the Loeb diagnostic/treatment criteria and the updated McGeer criteria were significantly lower in patients with impaired communication (p=0.005, 0.009, and 0.014, respectively), whereas rates of pyuria and positive urine cultures did not differ significantly. These findings suggest that asymptomatic bacteriuria may be more prevalent among patients with impaired communication than among verbal patients. Notably, antibiotics were prescribed to more than 98% of all patients whose urine sample was obtained.

**Table 4:** Comparison of the rate of meeting the criteria, urine tests, and antibiotic use between verbal patients and patients with impaired communication without indwelling catheter use *^1^ Urinalysis was not obtained in one case; *^2 ^Fisher’s exact test was used for pyuria and antibiotic use

Variables	Urinalysis of verbal patients (n=64)	Urinalysis of patients with impaired communication (n=24)	Test statistic	p-value
2005 Loeb diagnostic	35 (54.7%)	5 (20.8%)	χ^2^(1)=8.069	0.005
2005 Loeb treatment	27 (42.2%)	3 (12.5%)	χ^2^(1)=6.847	0.009
2012 updated McGeer criteria	32 (50.0%)	5 (20.8%)	χ^2^(1)=6.094	0.014
Pyuria (WBC>10)	57/63 (90.5%)*^1^	20 (83.3%)	*^2^	0.435
Positive urine culture (>10^5^CFU)	48 (75.0%)	16 (66.7%)	χ^2^(1)=0.611	0.434
Antibiotic use	63 (98.4%)	24 (100%)	*^2^	1.000

Table 5 shows the rate of meeting criteria, urine tests, and antibiotic use between verbal patients and patients with impaired communication who have indwelling catheters placed. There were no significant differences in the rates of meeting the three criteria between verbal patients and patients with impaired communication. There were a higher number of patients with impaired communication who met the three criteria than those without catheters. Likely because these criteria for catheterized patients include fewer subjective symptoms. In both groups of verbal patients and patients with impaired communication among catheterized patients, the rates of meeting the updated McGeer criteria were higher than those with positive urine cultures. There might be more false positives on the updated McGeer criteria among catheterized patients compared to patients without an indwelling catheter. As well as the non-catheterized group, antibiotics were prescribed to more than 90% of all patients whose urine sample was obtained. Table 6 shows which bacteria were detected in urine cultures, and the antibiotics used to treat these cases.

**Table 5:** Comparison of the rate of meeting the criteria, urine tests, and antibiotic use between verbal patients and patients with impaired communication with indwelling catheter use *^1^ Missing documentation about symptoms in three cases; *^2^ Fisher’s exact test was used for all variables

Variables	Urinalysis of verbal patients (n=27)	Urinalysis of patients with impaired communication (n=10)	p-value
2005 Loeb diagnostic	13/24 (54.2%)*^1^	5 (50.0%)	1.000*^2^
2005 Loeb treatment	13/24 (54.2%)*^1^	5 (50.0%)	1.000*^2^
2012 updated McGeer criteria	18/24 (75.0%)*^1^	8 (80.0%)	1.000*^2^
Pyuria (WBC>10)	26 (96.3%)	10 (100.0%)	1.000*^2^
Positive urine culture (>10^5^CFU)	15 (55.6%)	4 (40.0%)	0.476*^2^
Antibiotics use	26 (96.3%)	9 (90.0%)	0.473*^2^

**Table 6:** The bacteria detected in urine cultures and the antibiotics used (total n=127) IM: Intramuscular; IV: Intravenous; ESBL: Extended-spectrum beta-lactamase; MRSA: Methicillin-resistant *Staphylococcus aureus*

Organism	Colony count	n	Antibiotics used (no. of cases)
Escherichia coli	≥10⁵	21	Cephalexin (7), cefpodoxime (3), nitrofurantoin (3), IM ceftriaxone (5), sulfamethoxazole and trimethoprim (2), amoxicillin (1)
	≥10⁵ with another organism <10⁵	1	Cephalexin (1)
	<10⁵	6	Cefpodoxime (3), ciprofloxacin (1), cephalexin (1), nitrofurantoin (1)
Proteus mirabilis	≥10⁵	17	Cephalexin (8), cefpodoxime (2), sulfamethoxazole and trimethoprim (2), ciprofloxacin (2), IM ceftriaxone (2), meropenem (1)
	≥10⁵ with another organism <10⁵	1	IM ceftriaxone (1)
	<10⁵	3	Cefpodoxime (1), IM ceftriaxone (1), ertapenem (1)
Klebsiella pneumoniae	≥10⁵	16	Cephalexin (6), sulfamethoxazole and trimethoprim (4), cefpodoxime (3), nitrofurantoin (1), IM ceftriaxone (1), IV ertapenem (1)
	≥10⁵ with another organism <10⁵	1	Sulfamethoxazole and trimethoprim (1)
	<10⁵	10	Nitrofurantoin (3), IM ceftriaxone (3), cephalexin (1), sulfamethoxazole and trimethoprim (1), IV cefepime (1), no antibiotic used (1)
Pseudomonas aeruginosa	≥10⁵	2	Levofloxacin (1), ciprofloxacin (1)
	≥10⁵ with another organism <10⁵	1	Cefpodoxime (1)
	<10⁵	8	Ciprofloxacin (3), nitrofurantoin (2), amoxicillin and clavulanic acid (1), IM cefepime (1), IV meropenem (1)
*E. coli *(ESBL)	≥10⁵	2	Sulfamethoxazole and trimethoprim (1), cephalexin (1)
	≥10⁵ with another organism <10⁵	1	Nitrofurantoin (1)
	<10⁵	1	Doxycycline (1)
Enterococcus faecalis	≥10⁵	3	Nitrofurantoin (2), cefpodoxime (1)
	<10⁵	1	Nitrofurantoin (1)
Staphylococcus aureus	≥10⁵	2	Clindamycin (1), ciprofloxacin (1)
	≥10⁵ with another organism <10⁵	1	Cefpodoxime (1)
MRSA	≥10⁵	1	Cephalexin (1)
	<10⁵	1	No antibiotic used (1)
Providencia stuartii	≥10⁵	1	Sulfamethoxazole and trimethoprim (1)
	<10⁵	1	Amoxicillin and clavulanic acid (1)
Other organisms (≥10⁵)	Aerococcus sanguinicola	1	Cephalexin (1)
	Enterobacter cloacae	1	Cefpodoxime (1)
	Klebsiella oxytoca	1	Nitrofurantoin (1)
	*K. oxytoca *with another organism <10⁵	1	Cephalexin (1)
Other organisms (<10⁵)	Aerococcus urinae	1	No antibiotic used (1)
Two organisms ≥10⁵	*P. mirabilis* and *P. aeruginosa*	3	Cephalexin (2), ciprofloxacin (1)
	*P. mirabilis *and *P. stuartii*	1	Doxycycline (1)
	*E. coli *and* P. stuartii*	1	Sulfamethoxazole and trimethoprim (1)
	*E. coli *and *A. sanguincola*	1	IM ceftriaxone (1)
	*K. pneumoniae* and *P. stuartii*	1	IV ertapenem (1)
	*Proteus valgaris *and *Morganella morganii*	1	IM ceftriaxone (1)
More than three organisms		3	Cefpodoxime (1), nitrofurantoin (1), no antibiotic used (1)
Mixed flora		6	Nitrofurantoin (2), cephalexin (1), cefpodoxime (1), sulfamethoxazole and trimethoprim (1), IM ceftriaxone (1)
No growth		2	Levofloxacin (1), IM ceftriaxone (1)
Urine culture not collected		1	Doxycycline (1)

## Discussion

Our research examined the disparity in urinalysis and urine culture outcomes between verbal patients and patients with impaired communication. The findings indicated a notable discrepancy in meeting Loeb (diagnostic/treatment) and updated McGeer criteria among patients with impaired communication in the non-catheterized cohort, with significantly lower rates observed. Additionally, a high prevalence of antibiotic prescriptions was noted among patients from whom urine samples were collected.

Our investigation noted a disparity in the rates at which patients with impaired communication and verbal patients met these diagnostic criteria. This discrepancy may stem from several factors, including the lower manifestation of typical UTI symptoms in patients with impaired communication and the decreased prevalence of such symptoms in older adults, prompting healthcare providers to procure urine samples. Notably, patients with cognitive impairment often struggle to articulate UTI symptoms listed in the Loeb criteria, such as urgency, frequency, or suprapubic pain, and favor nonspecific symptoms such as confusion instead of diffuse abdominal pain. Moreover, older adults typically exhibit fewer typical UTI symptoms, such as dysuria and frequency [11]. In our study, urine samples were frequently obtained from patients with impaired communication due to nonspecific symptoms, such as confusion. This pattern may reflect clinician bias, with a tendency to order more diagnostic tests for patients with impaired communication, potentially influenced more by the patients’ cognitive status than by objective clinical criteria.

Currently, there is insufficient evidence to accurately associate UTI with confusion [12,13]. The American Medical Directors Association's (AMDA) Infection Advisory Subcommittee acknowledged the challenge in diagnosing UTIs in patients unable to verbalize their symptoms, but no specific recommendations were provided for determining genitourinary-specific symptoms in patients with impaired communication [14]. Similarly, a review published in the Journal of the American Geriatric Society highlighted the difficulty in ascertaining symptoms among LTC facility residents due to impaired communication and functional disability, yet it did not offer guidance on managing UTI symptom ascertainment in patients with impaired communication [15]. Given these observations, we contend that the Loeb diagnostic criteria may be overly stringent for patients with impaired communication, hindering accurate differentiation between UTI and asymptomatic bacteriuria. 

The findings revealed a high antibiotic utilization rate exceeding 90% across all patients, alongside significantly lower rates of meeting the Loeb treatment criteria among patients with impaired communication. Potential reasons for high antibiotic prescription rates include difficulty in identifying classic UTI symptoms among patients with impaired communication and fear of missing severe infections, such as sepsis. The high antibiotic prescription rates are similar to those of D'Agata et al.'s study, which reported that 75% of prescriptions for UTI in LTC facility residents were given to individuals who did not meet the criteria for UTI [16]. This trend significantly contributes to inappropriate antimicrobial use within LTC facilities, fostering antimicrobial resistance and *Clostridium difficile* infection among residents [16,17]. Reducing unnecessary urine tests is imperative based on our findings. Moreover, a separate study indicated that treatment failed to enhance activities of daily living scores at two or seven days, suggesting that urine culture testing in LTC facilities does not yield functional improvements [18]. As urine culture testing and treatment do not lead to functional improvement, restricting access to the test may be reasonable.

Strengths and limitations

This study provides insights into real-world clinical practice patterns of urine testing and antibiotic use in an LTC setting, focusing on a vulnerable population with impaired communication. By applying established diagnostic criteria (Loeb and updated McGeer), it highlights important disparities between verbal patients and patients with communication impairment, contributing to the understanding of challenges in diagnosing UTIs in cognitively impaired residents. Additionally, the inclusion of a full year of data and total population sampling strengthens the reliability of the findings within the study setting.

Our study possesses several limitations. First, being a single-center observational study, its findings may lack generalizability. Nonetheless, we contend that our study retains a degree of universality, as the results align with prior research and contribute to the evaluation of disparities between verbal patients and patients with impaired communication. Second, our analysis did not capture correlations between the results and the detailed subtypes or underlying causes of impaired communication, which limited our ability to assess how specific communication barriers might differentially influence testing and prescribing patterns. Third, unmeasured confounders, such as variations in clinician judgment due to differences in providers' education, differences in the quality of sampling techniques, or patient-level factors not captured in our dataset, may have influenced our findings. Regarding sampling technique, although we did not directly observe all specimen collection procedures, we believe poor technique was unlikely to have caused many cases of contamination, as no data indicated that samples collected by a particular nurse or from a specific floor had significantly higher rates of urine culture results suggestive of contamination. Finally, our study did not assess adverse events such as multiple drug resistance or *C. difficile* infection. Further prospective studies are required, and additional research is warranted to evaluate individual risks associated with antibiotic usage or urine tests. 

## Conclusions

The present study revealed that patients with impaired communication exhibited lower rates of meeting both the Loeb diagnostic and treatment criteria than verbal patients. This discrepancy is likely attributable to the inclusion of multiple subjective symptoms in the Loeb diagnostic criteria, which pose challenges for healthcare providers to assess accurately in patients who cannot effectively communicate their symptoms. Discrepancies between updated McGeer criteria adherence and positive culture rates suggest urine tests are often ordered for asymptomatic bacteriuria in this patient group. Further research is needed to develop and implement a modified diagnostic algorithm tailored to the unique clinical presentation of LTC residents with impaired communication to distinguish true UTIs from asymptomatic bacteriuria more accurately. Additionally, our findings emphasize the importance of reducing unnecessary urine testing to mitigate the unwarranted use of antibiotics. 
